# Supplementary material for: Probabilistic Human Health Risk Assessment of Inorganic Arsenic Exposure Following the 2020 Taal Volcano Eruption, Batangas, Philippines
Source: Toxics. 2025 Dec 22;14(1):13. doi: 10.3390/toxics14010013 (PMC12845767; doi:10.3390/toxics14010013)
Supplement: Supplementary file 1 [file toxics-14-00013-s001.zip › toxics-4030816-supplementary.pdf]

Probabilistic Human Health Risk Assessment of Inorganic Arsenic Exposure Following the 2020 Taal Volcano Eruption, Batangas, Philippines

Yu-Syuan Luo[1,2,3,*], Jullian Patrick C. Azores[4], Rhodora Reyes[5,*], Geminn Louis C. Apostol[6]

[1]Master of Public Health Program, College of Public Health, National Taiwan University, Taipei City, Taiwan

[2]Institute of Food Safety and Health, College of Public Health, National Taiwan University, Taipei City, Taiwan
[3]Population Health Research Center, College of Public Health, National Taiwan University, Taipei City, Taiwan
[4]Department of Physical Sciences and Mathematics, University of the Philippines Manila, Manila City, 1000 Philippines
[5]Philippine Neurology Association, Fellow Philippine Society of Clinical and Occupational Toxicology, Consultant National Toxicology Specialty
[6]School of Medicine and Public Health, Ateneo de Manila University, Don Eugenio Lopez Sr. Medical Complex, Ortigas Ave, Pasig 1604, Philippines

Item	Title
**Supplemental Table S1**	Total arsenic concentrations in clams collected from Taal lake in 2023.
**Supplemental Table S2**	Morphometric Characteristics, Moisture Content, and Dry-to-Wet Conversion Factors of Corbicula fluminea (White and Purple Morphotypes) Collected from Three Harvesting Sites in Taal Lake (Saluyan, Calauit, and Binintiang Munti), Batangas, Philippines

Supplemental Table S1. Total arsenic concentrations in clams collected from Taal lake in 2023.

File #	DateTime	Application	Method	As	Multiplier	Cal Check	
1292	04-27-2023 15:31	GeoExploration	Oxide3phase	**0.0006**	4	Passed	0.0006
1293	04-27-2023 15:34	GeoExploration	Oxide3phase	**0.0005**	4	Passed	0.0005
1294	04-27-2023 15:36	GeoExploration	Oxide3phase	**0.0007**	4	Passed	0.0007
**Site:**	Saluyan	**Morph**	White				
**Parts per million (ppm)**				**6±3**			0.0006
1295	04-27-2023 15:39	GeoExploration	Oxide3phase	**0.0006**	4	Passed	0.0006
1296	04-27-2023 15:41	GeoExploration	Oxide3phase	**0.0006**	4	Passed	0.0006
1297	04-27-2023 15:43	GeoExploration	Oxide3phase	**0.0005**	4	Passed	0.0005
**Site:**	Saluyan	**Morph**	Purple				
**Parts per million (ppm)**				**5.67±3**			0.000566667
1298	04-27-2023 15:45	GeoExploration	Oxide3phase	**0.0004**	4	Passed	0.0004
1299	04-27-2023 15:47	GeoExploration	Oxide3phase	**0.0005**	4	Passed	0.0005
1300	04-27-2023 15:49	GeoExploration	Oxide3phase	**0.0006**	4	Passed	0.0006
**Site:**	Calauit	**Morph**	White				
**Parts per million (ppm)**				**5±3**			0.0005
1301	04-27-2023 15:51	GeoExploration	Oxide3phase	**0.0007**	4	Passed	0.0007
1302	04-27-2023 15:53	GeoExploration	Oxide3phase	**0.0005**	4	Passed	0.0005
1303	04-27-2023 15:55	GeoExploration	Oxide3phase	**0.0005**	4	Passed	0.0005
**Site:**	Calauit	**Morph**	Purple				
**Parts per million (ppm)**				**5.67±3**			0.000566667
1304	04-27-2023 16:11	GeoExploration	Oxide3phase	**0.0007**	4	Passed	0.0007
1305	04-27-2023 16:12	GeoExploration	Oxide3phase	**0.0006**	4	Passed	0.0006
1306	04-27-2023 16:18	GeoExploration	Oxide3phase	**0.0006**	4	Passed	0.0006
**Site:**	Binintiang Munti	**Morph**	White				
**Parts per million (ppm)**				**6.33±3**			0.000633333
1307	04-27-2023 16:21	GeoExploration	Oxide3phase	**0.0006**	4	Passed	0.0006
1308	04-27-2023 16:23	GeoExploration	Oxide3phase	**0.0005**	4	Passed	0.0005
1309	04-27-2023 16:25	GeoExploration	Oxide3phase	**0.0005**	4	Passed	0.0005
**Site:**	Binintiang Munti	**Morph**	Purple				
**Parts per million (ppm)**				**5.33±3**			0.000533333

**As Err**			
0.0003	0.0003		0.000300
0.0003	0.0003		
0.0003	0.0003		
0.0003	0.0003		0.000300
0.0003	0.0003		
0.0003	0.0003		
0.0003	0.0003		0.000300
0.0003	0.0003		
0.0003	0.0003		
0.0003	0.0003		0.000300
0.0003	0.0003		
0.0003	0.0003		
0.0003	0.0003		0.000300
0.0003	0.0003		
0.0003	0.0003		
0.0003	0.0003		0.000300
0.0003	0.0003		
0.0003	0.0003		

Supplemental Table S2. Morphometric Characteristics, Moisture Content, and Dry-to-Wet Conversion Factors of Corbicula fluminea (White and Purple Morphotypes) Collected from Three Harvesting Sites in Taal Lake (Saluyan, Calauit, and Binintiang Munti), Batangas, Philippines

Locality	Species	Harvest weight (g)	Wet weight (W_{wet}) (g)	ry weight (W_{dry}) (g	Moisture content (Pr_{water}	nversion factor (CF) (dry/w	Average length(cm)
Saluyan (S)	White		415	55.49	86.63%	0.1337	22.49
	Purple	5280	240	27.15	88.69%	0.1131	20.05
Calauit (C)	White		480	59.37	87.63%	0.1237	23.68
	Purple	5190	270	33.09	87.74%	0.1226	21.13
Binintiang Munti (BM)	White		410	48.43	88.19%	0.1181	21.03
	Purple	5000	270	35.82	86.73%	0.1327	19.00

	S length		C length		BM length	
	White	Purple	White	Purple	White	Purple
1	21.4	17.7	24.4	26.6	25.45	17.75
2	21.8	23.7	23.05	18	23.1	23.2
3	20.85	18.8	19.75	18.8	18.05	19.3
4	25.6	16.6	22.95	19.65	26.1	20.4
5	26.35	17.45	21.6	27.45	25.3	22.7
6	19.95	19.45	27.4	26.9	18	24.4
7	24.7	27.6	25.85	21.55	20.25	20.25
8	23.2	18.8	26.85	20.75	20.8	20.15
9	24.05	21.35	25.5	23.55	18.8	17.05
10	23.95	23.3	18.95	17.7	22.7	18.3
11	16.55	16.9	21.85	17.85	23	16.3
12	22.45	22.2	23.7	20.2	24.75	17.35
13	26.5	22.1	20.55	24.1	16.6	21.2
14	20.55	16.6	22.85	20.5	22.05	20.2
15	18.35	19.6	27.2	20.25	11.6	19.4
16	24.45	18.8	27.5	20.6	22.9	17.45
17	24.6	16.85	21.3	22.5	21.15	21
18	17.5	20.9	27.45	21.6	14.9	17.85
19	21.5	24.4	26	19.6	24.3	21.3
20	30.2	19.05	24.95	22.7	17.3	15.25
21	28	18.8	21.85	23.65	23.8	20.8
22	25.85	19.1	24.4	17.45	23.55	19.7
23	16.9	19.8	22.4	19	15.9	16.5
24	24.6	22.45	25.25	20.5	23.3	18.05
25	25.5	17.4	19.85	28.55	21.6	17.35
26	22.45	20.75	24.85	19.9	25.85	20
27	21.3	22.2	19.3	18.9	22.45	17.5
28	26.85	18.8	21.6	20.15	18.55	18.55
29	12.45	17.8	25.3	17.3	19.9	15.5
30	16.25	22.25	25.8	17.7	18.8	15.3
